# Vaccines against leishmaniasis: using controlled human infection models to accelerate development

**DOI:** 10.1080/14760584.2021.1991795

**Published:** 2021-10-27

**Authors:** Vivak Parkash, Paul M. Kaye, Alison M Layton, Charles J Lacey

**Affiliations:** a York Biomedical Research Institute, Hull York Medical School, University of York, York, UK; b Department of Infection and Tropical Medicine, Sheffield Teaching Hospitals Nhs Foundation Trust, Sheffield, UK

**Keywords:** Controlled human infection model (CHIM), human challenge, leishmaniasis, vaccines, experimental models, experimental infection

## Abstract

**Introduction:**

Leishmaniasis is a neglected tropical disease that is defined by the World Health Organization as vaccine preventable. Although several new candidate vaccines are in development, no vaccine has successfully reached the market for human use. Several species of *Leishmania* cause human disease and have co-evolved with their respective sand fly vectors. These unique relationships have implications for initiation of infection and vaccine development. An approach to vaccine development for many infectious diseases is the use of controlled human infection models (CHIMs).

**Areas covered:**

We describe the history and recent development of experimental and deliberate infection using *Leishmania* in humans and the rationale for developing a new sand fly-initiated CHIM to progress leishmaniasis vaccine development. Examples from other infectious diseases are discussed in the context of the development of a new leishmaniasis CHIM. We also reflect upon the manufacture of the challenge agent, practical considerations, safety, ethics, and regulatory issues.

**Expert opinion:**

A new cutaneous *Leishmania* CHIM is being developed to enable testing of vaccines in the development pipeline. Questions remain about the use of such CHIMs to determine effectiveness of vaccines against visceral leishmaniasis. However, such a CHIM will be invaluable in expediting time to market for vaccines.

## 1. Background

### 1.1. Understanding the problem and the clinical context of leishmaniasis

Leishmaniasis is a neglected tropical vector-borne disease caused by infection with the intracellular protozoan parasite *Leishmania*. It is endemic in at least 98 countries across the world [1], with over 50 species of *Leishmania* having been discovered [2], of which 20 species, belonging to two subgenera, having the potential to cause human disease [3]. Over 100 million people are deemed to be at risk of contracting leishmaniasis, with estimates suggesting 1 billion people reside in endemic regions [1, 4–6]. Up to 1 million new cases occur each year [6], with an estimated 20,000 deaths, representing a significant burden of disease [7]. Leishmaniasis is largely a disease of poverty and areas of endemicity show over-representation by low- and middle-income (LMIC) countries. Leishmaniasis causes a spectrum of disease, predominantly affecting the skin (tegumentary leishmaniasis), but with potential to cause fatal systemic disease (visceral leishmaniasis; VL). The range of skin manifestations are broad including localized or widespread lesions with or without mucosal involvement. Disease pathogenesis is determined predominantly by the species of infecting *Leishmania* parasite, with distinct geographical regions associated both with particular parasite and vector species and specific clinical manifestations, with the prefix ‘Old World’ (reflecting the Middle East, Africa, and Asia) and ‘New World’ (reflecting the Americas) often being employed. Host genetics and environmental factors also play an important role [8]. Elimination campaigns in South Asia have helped decrease the numbers of new cases of VL although substantial barriers remain [9]. Countries bordering the Mediterranean are also endemic for leishmaniasis, and sporadic cases not linked to travel have been reported in non-endemic settings [10]. The natural vector is the female phlebotomine sand fly, which becomes infected as a result of ingestion of parasites when taking a blood meal. Over 700 species of sand fly have been discovered to-date [2], and close to 100 are vectors of *Leishmania* that can cause human disease [3]. *Leishmania*-associated sand flies are geographically distributed in mainly tropical climates, although species are found in temperate climates which has implications for disease spread and immunity [11].

### 1.2. The burden of disease and issues affecting clinical management

The most commonly reported form of leishmaniasis is localized cutaneous leishmaniasis (CL). Typically, this results in skin lesions that have the ability to self-heal, with or without ulceration. Causative species include *L. major* and *L. tropica* in the Old World, and the *L. mexicana* complex and the *L. (Viannia) braziliensis* complex in the New World. New World species may also result in mucosal leishmaniasis (*L. (Viannia) braziliensis*), diffuse cutaneous leishmaniasis (*L. amazonensis)* and disseminated cutaneous leishmaniasis (*L. (Viannia) guyanensis*). *Leishmania aethiopica* infection can produce the entire spectrum of tegumentary disease in Ethiopia [12]. Visceral leishmaniasis (VL), also known as kala-azar, is caused by parasites of the *L. donovani* complex. It affects the internal organs and is usually fatal if left untreated. Symptoms include organomegaly and end-organ damage, bone marrow suppression, cachexia and anorexia, persistent fever as well as an increased incidence of secondary infections [13,14]. In total, 90% of VL cases are reported from Brazil, Ethiopia, India, Kenya, Somalia, South Sudan and Sudan, with 70% of cases in India reported from a single state, Bihar [15]. VL transmission is typified in the Indian subcontinent by anthroponotic transmission, though recent reports suggest a possible canine reservoir [16]. In contrast, in the Americas, VL is typically zoonotic. Following treatment, up to 20% of patients may develop post-kala-azar dermal leishmaniasis (PKDL), characterized by a progressive maculopapular rash, which can transform into nodular eruptions [17]. PKDL is thought to be predominantly immunologically mediated [18], linked to persistent parasites in the skin lesions and patients remain infectious for sand flies [17, 19, 20]. HIV coinfection worsens the prognosis for both VL and PKDL patients [21,22].

A limited number of effective anti-leishmanial drugs are available (reviewed elsewhere [23–25]) but risks of drug resistance remain and standard care has limited efficacy in HIV co-infected patients. Currently no vaccines have been licensed for use in the prevention of human leishmaniasis, though considerable progress has been made in recent years and it is generally accepted that a vaccine to prevent one or all forms of leishmaniasis would have considerable public health value.

## 2. Controlled human infection models

### 2.1. A background to studies of controlled human infection

Controlled human infection model (CHIM) studies use deliberate methods of exposure of human participants to pathogens, most commonly to test efficacy of candidate vaccines or to understand disease pathogenesis. Many terms are used interchangeably to describe controlled deliberate infection studies on human participants. Discussion within the field, evidenced by a workshop held by the Academy of Medical Sciences in 2018 [26], has strengthened the use of the term ‘controlled human infection models’ (CHIM), as most accurately and uniformly describing these studies. The importance of uniformity of language surrounding such studies is important to ensure clarity of message and avoidance of confusion, particularly as such studies are becoming increasingly well known outside of science-literate audiences. The term ‘challenge’ is used commonly in reference to these studies, with similar terms such as ‘human challenge models/studies/trials,’ ‘human infection challenge,’ ‘microbial challenge studies,’ ‘volunteer infection studies,’ amongst many other variations. Infectious agents used in CHIM studies are most commonly referred to as the ‘challenge agent.’

Using CHIM studies to measure efficacy of interventions at an early stage of development has a number of advantages when coupled with traditional vaccine studies and immunological analyses. In particular, immunological correlates of protection can be derived, whilst candidate vaccines can be up- or down-selected at an early stage of development. This can also be more cost-effective in preventing potentially poorly performing candidates heading into large scale clinical trials. As such, resources can be more effectively distributed in supporting a larger number of candidate vaccines, enhancing the possibility of a successful vaccine being developed. However, the predictive value of CHIMs should always be considered, particularly when there is significant divergence between disease progression and/or endpoints in a CHIM compared to the disease course in a real-world endemic setting.

CHIM studies have contributed significantly to our understanding of many diseases and attempts at control, with new vaccines being tested, novel discoveries and more accurate translation of results as compared to animal studies. Many such CHIMs and prospective human infection studies exist for a multitude of diseases, described in depth elsewhere [27–35]. Many studies using CHIM models have generated meaningful new data, even where they have either failed to demonstrate efficacy of an intervention or where testing of specific treatments has not taken place. For example, in prospective studies involving dengue virus, CHIM studies provided valuable insight into transformation to dengue hemorrhagic fever, and a source of serial peripheral blood mononuclear cells (PBMC), sera and plasma that may prove valuable for identifying determinants of disease, correlates of protection and other downstream comparative immunological analyses [31]. In a CHIM study for *Streptococcus pneumoniae*, it was shown that carriage of pneumococcus was functionally significant and protected against subsequent carriage. This could not be determined purely by using animal models. Furthermore, this CHIM allowed the pathogen dose dependency of the antibody responses to be determined [32]. In a norovirus CHIM, it was found that infected immunocompetent hosts do not display a measurable viremia[30]. Archived material from this early norovirus challenge study has contributed to novel research findings in more recent studies, with implications for norovirus diagnostics and assays [36]. In a CHIM for schistosomiasis, although the impact of repeated infection in endemic settings was not reproduced, the utility of early diagnostic tests could be confirmed [33]. A CHIM study for malaria indicated that strong cellular immune responses did not impact on parasite growth rates, leading to a focus on achieving sufficient antibody titers[37]. In some cases, CHIM studies have been particularly beneficial where there is a lack of validated animal models. This is particularly true of dengue virus, where non-human primates do not display clinical disease despite measurable viremia [31,38]. CHIM studies are even being considered for many diseases that had previously been thought to have been ‘unchallengeable’ due to ethical and pathological reasons, such as experimental infection with bacillus Calmette-Guérin (BCG) as a surrogate for *Mycobacterium tuberculosis* [39]. The use of CHIMs for rapid development and testing of emerging viral pathogens has been realized with a CHIM for SARS-CoV-2 currently being investigated [35].

### 2.2. *The history of experimental* Leishmania *infection and sand fly bite on human participants*

The early part of the 20^th^ century bought with it an abundance of small experiments in quick succession that improved the understanding of the disease, the parasite and mode of transmission. The first documented experimental transmission to humans was in 1907, using subcutaneous inoculation [40]. Further work suggested the association of the *Leishmania* parasite with the sand fly [41]. The role of the sand fly in transmission of the parasite was then confirmed in experiments on human participants, although these early investigations did not expose humans directly to sandflies [42]. The first documented human exposure to sand flies in an experimental setting involved xenodiagnosis to confirm transmission to non-infected sand flies from infected human participants [43]. Further pioneering work by Adler and Theodor in the early 20^th^ century successfully confirmed the transmission of *Leishmania* from phlebotomine sand flies directly to humans and this has been reproduced since, as well as many experimental human infections using parenteral methods [44–51]. This early work elucidated the life cycle of the *Leishmania* parasite in the sand fly and the finding of transmission via sand fly bite was made. It was also demonstrated that artificial feeding on infected tissue was comparable to transmission from human to sand fly [52,53]. Subsequent studies demonstrated transmission using different sand fly and *Leishmania* species [54–56]. Inoculation of *Leishmania infantum*, causing canine leishmaniasis, into human participants, which demonstrated the ability for similar *Leishmania* species to infect and manifest similar disease phenotypes across mammalian species, confirmed the suggestions of an infectious reservoir across species with relevance to human disease [57].

A long-standing practice for many centuries in *Leishmania*-endemic countries, particularly in the Middle East, was ‘leishmanization.’ This involves inoculation of parasites transferred from a person with an active cutaneous lesion, to an anatomical site where lesion development and scarring would be less stigmatizing such as the buttocks. The observation was made that healing of the lesion protected the recipient from further *Leishmania* infections throughout their lifetime [58]. Leishmanization proliferated in the mid to later part of the 20^th^ century, notably involving service personnel involved in conflicts in hyperendemic areas of the Middle East. Approximately 2 million people underwent leishmanization in this setting, which included a number of refugees, and a significant reduction in new cases was observed [59]. After the cessation of the Iran–Iraq conflict, leishmanization was largely abandoned. Adverse effects included exacerbated localized reactions in comparison to the usual disease course of the inoculated species *Leishmania major* [60], and hypersensitivity [59]. Reliability and viability of the inoculated parasite also varied widely, although currently leishmanization still occurs sporadically in Uzbekistan [61].

More recently, the practice of leishmanization has been studied further to assess reproducibility and utility to study new vaccines and indeed for use as a potential live vaccine [44, 62–65]. This has been facilitated in part by improved parasite culture techniques [66] and improved understanding of good manufacturing practices (GMP). Contemporary experimental infection studies have also sought to examine the immunological basis of infection. One study attempted to demonstrate cross-protection using exposure to *L. arabica*-infected *P. papatasi* in one human participant, and needle challenge in 3 participants [67]. The participant challenged by sand fly bite did not develop lesions, and all other participants developed lesions by day 250 after subsequent *L. major* challenge. A further study, using an *L. major* preparation made under GMP, demonstrated lesions in 19 out of 23 healthy *Leishmania*-naïve participants, using needle challenge[63]. The majority (74%) developed ulcerated lesions by day 60, although all healed successfully, either spontaneously or with treatment. Collectively, these historic human infection studies have laid the groundwork for developing a new CHIM that builds on recent advances in our understanding of transmission.

## 3. Developing a CHIM for leishmaniasis

### 3.1. Pathway to an effective CHIM

Numerous non-human mammalian models exist for the study of both cutaneous and visceral forms of disease, which have added significant insight in to the behavior of host and pathogen [68, 69]. However, the predictive power of these animal models in terms of human response has been called into question [70]. Mice are most commonly used for in vivo experimental *Leishmania* studies, although different strains of mice have distinct immunological responses to *Leishmania* infection and therefore translation to clinical practice may be difficult to predict [71, 72], emphasizing the value of direct studies in humans.

Observations from CHIMs of other infectious diseases has suggested that the threshold for developing a CHIM is multi-fold: poor disease control and impact on morbidity and mortality, lack of successful vaccines, a number of candidate vaccines in the development pipeline and/or potential candidate antigens, absence of effective treatments and/or evidence of drug resistance, and perhaps most important a treatable strain or species of the infectious agent relevant to clinical disease. Given the frequent lack of resources and economic constraints in the endemic settings where infectious diseases occur, pilot studies to develop CHIMs are often conducted in high-income countries (HIC), with subsequent rollout to endemic settings. As the regulatory and ethical issues around CHIMs become more established, this trend may reverse, providing an additional route for long-term research capacity building in LMICs and facilitating the development of CHIMs adapted to local settings.

**Figure 1.**
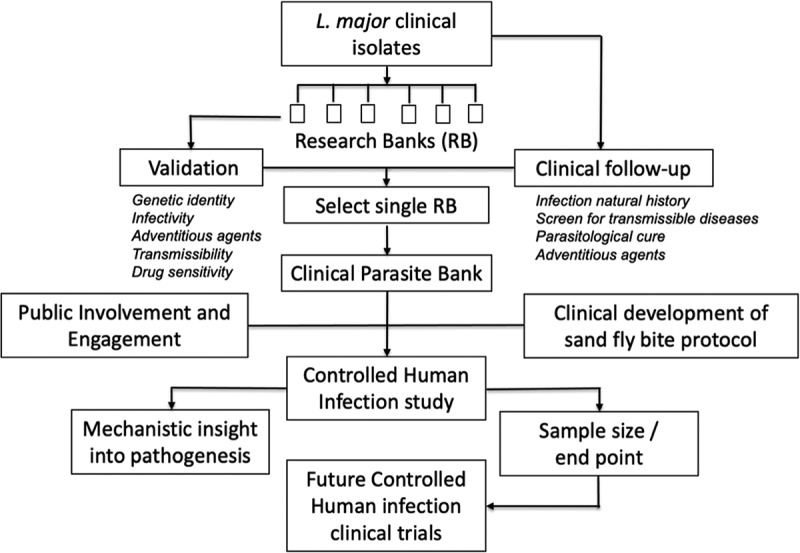
Potential development process for a cutaneous leishmaniasis CHIM

Given the established feasibility of experimental human infection with leishmaniasis using both needle challenge and sand fly-initiated infection, a first step in developing a new CHIM is to ensure that it is effective, reproducible and safe for participants. As such a series of enabling studies and exercises are required, including establishing a well characterized and ideally GMP compliant parasite bank for clinical use, with validation of the effectiveness and acceptability of sand fly bite and public engagement to ensure transparency, accountability and acceptability (see Figure 1).

### 3.2. Public involvement and engagement

Public engagement and involvement is now embedded within healthcare and research practice in the UK [73] and elsewhere. Given the demonstrable impact of public involvement in driving improved study design, recruitment and retention and scientific outcomes, public involvement has now become a necessity for many funders [74]. Public involvement in CHIMs is particularly pertinent of late, with discussion of the acceptability of a SARS-CoV-2 CHIM to help control the COVID-19 pandemic [75]. There is also significant breadth of understanding of CHIM studies by the public in LMICs [76], and public engagement is therefore an important consideration when conducting novel CHIM studies in endemic studies, even where prior consultation has taken place [77].

There are limited studies that assess public awareness of issues concerning leishmaniasis [78], and until recently no such studies at the intersection of CHIMs and leishmaniasis. Parkash et al. recently described a public involvement study related to the development of a cutaneous leishmaniasis CHIM using infected sand flies [79]. Ten members of the public, including a National Institute for Health Research (NIHR) patient research ambassador and a previous CHIM study participant were recruited to discuss the basis of a *Leishmania* CHIM, in a focus group chaired by a health psychologist. The study design of a proposed CHIM and underpinning rationale from a scientific, cost-effectiveness and ethical basis were discussed. Participants were invited to review draft study documentation prior to their involvement. Common themes were identified and analyzed to identify the importance to both investigators and participants, separated into 1) the accuracy and accessibility of written participant-facing material, 2) study design and 3) motivation(s) for involvement in the proposed research. In terms of the written material, participants made it clear that plain English summaries of research are important to engage participants and the public more broadly. Specific to a *Leishmania* CHIM, participants suggested that given the potential for anaphylaxis or hypersensitivity from *Leishmania*-infected sand fly bite, it was important to explicitly discuss this in the written material, quantifying risk and explaining mitigations. Input from participants around the theme of study design suggested scarring was an acceptable clinical consequence from a cutaneous leishmaniasis lesion, if it remained localized and limited in size and a choice of anatomical site was given. Participants were presented with a range of possible treatment options used more broadly for leishmaniasis. Of these, early excision of a lesion(s) was an unexpected choice favored by participants. The motivations given for this choice being both the altruistic aspect of donating tissue for analysis to gain potential new insights into pathogenesis as well as a psychological component that the procedure may ‘remove’ the infection. This activity subsequently informed the development of a study protocol to develop a CHIM [79, 80]. Unfortunately, no participant who had previously undergone treatment for leishmaniasis was able to be recruited for this public engagement study.

### 3.3. Regulatory compliance

Regulatory issues will reflect the setting in which a CHIM is to be conducted. In the UK for example, all studies involving an Investigational Medical Product (IMP), are regulated by the Medicines and Healthcare products Regulatory Agency (MHRA). An infectious challenge agent pertaining to any CHIM study is, however, not deemed to be an IMP and is therefore not bound by regulations set out by the MHRA. A proposed UK study to develop a new CHIM using *L. major*, does not therefore require regulatory approval from the MHRA [80]. However, if an IMP is to be used within the context of a CHIM study, for example, a vaccine trial, then the MHRA would give due consideration to the nature of the challenge agent in addition to its existing obligation to regulate any IMP. In general, in the UK, it is expected that all clinical trial conduct is nevertheless carried out in accordance with Good Clinical Practice (GCP). In some regions an infectious challenge agent is required to be regulated, as per any vaccines or other IMP, such as in the US [81]. In regions where there is lack of formal guidance specifically for conduct of CHIMs, the relevant regulatory agency concerning IMPs should be consulted for advice.

CHIM specific guidance varies widely by region, and no formal regulatory guidance yet exists in the UK. However, in 2016 the World Health Organization released a position statement on regulatory considerations for CHIM studies [81]. This sets out some broad considerations for conduct of CHIM studies related specifically to the infectious challenge agent, appropriateness of the use of CHIM studies, design considerations, potential infrastructure and operational features and ethical considerations. The establishment of CHIMs in endemic country settings may face additional barriers, including regulatory capacity, risk assessment and clinical governance, as exemplified in the establishment of a CHIM for schistosomiasis in Uganda [77].

### 3.4. Safety & ethical considerations

Subsequent to any necessary local regulatory approval, ethical approval must be sought through a research ethics committee, which in the UK is conducted by the Healthcare Regulatory Authority (HRA). Ethical review is imperative to CHIM studies, to ensure ethical standards are upheld and research participants are protected from intended and unintended harms. Some negative connotations with regard to deliberate human exposure to infectious disease remain, as a result of unethical practices in the early part of the 20^th^ century. In the last 50 years, improved application and research techniques as well as development of informed consent of participants have advanced their use, although ethical review is still imperative to secure public confidence in such studies. Several safety concerns exist with any proposed *Leishmania* CHIM, as with every other CHIM study, given the nature of deliberate infection. These concerns should form the basis of ethical review. A recent non-infected sand fly biting study navigated some of these issues [82], in preparation for a proposed CHIM for *Leishmania*, which has now received full approval by a UK NHS research ethics committee [80]. The mitigations for these perceived risks are discussed here. However, CHIMs in endemic settings may have additional ethical issues to overcome [83,84].

Anaphylaxis has been reported from some biting and hematophagous insects, although no cases of anaphylaxis have been linked to phlebotomine sand flies. There is, however, evidence to suggest delayed-type hypersensitivity against some species of phlebotomine sand fly [85]. In Australasia, the use of the term ‘sand fly’ encompasses non-phlebotomine flying insects that are associated with anaphylaxis and severe bite reaction [86,87]. Although these adverse effects associated with non-phlebotomine sand flies are not directly relevant, evidence from public engagement work has suggested that such connotations can impact on recruitment if not accounted for [79]. In a recent non-infected sand fly biting study, mitigations included robust exclusion criteria, including history of atopic disease, history of exaggerated response to insect bite and history of anaphylaxis. Immunosuppressed individuals were also excluded based on experience of leishmaniasis with immune altering conditions such as HIV, but also observations of the use of leishmanization and associated prolonged lesions related to immunosuppressed individuals [60]. Clinicians also sought to ensure availability of adequate resources to deal with anaphylaxis and other life-threatening conditions, which included a defibrillator, high-flow oxygen device and parenteral adrenaline, as well as the presence of trained clinical staff during proceedings.

A number of solicited reactions following a sand fly bite are anticipated, including itch, pain, erythema, swelling and potential blister formation at the bite site. In the recent study using uninfected sand fly bites, no serious adverse events (SAEs) were reported. A clinician-recorded grading system was also utilized for recording of objective adverse events, with a small number of recorded events at grade 2 out of a possible 4, with the majority of events being graded as 1 (Grade 1 – mild, Grade 2 – moderate, Grade 3 – severe, Grade 4 – extreme). Participants were encouraged to record symptomology using a daily diary card up to 21 days post-sand fly bite exposure. A 10-point self-reported visual analogue scale was used to record local effects at the bite site such as itch, pain/discomfort, erythema, swelling and blistering. Local effects were mild with mean score of between 0 and 1 up to day 21; localized erythema, swelling and itch were reported most frequently. Systemic events such as headache, malaise, myalgia and fever were also recorded and where reported were mild and attributable to 2 participants who had an intercurrent viral infection.

In experimental models, *Leishmania* persistence after clinical cure is well known and contributes to both long-term concomitant immunity to reinfection and reactivation of clinical disease. This is also observed with both HIV co-infection [21] and elective immunosuppression [88] in humans, and suggests the persistence of parasite of at least some *Leishmania* species. Hence, the likelihood of parasite persisting after termination of a CHIM study and the long-term consequences for volunteers must be considered. Comprehensive literature reviews identify multiple reports associated with *L. donovani, L. infantum* and *L. tropica* and with various New World species causing tegumentary disease [89,90]. In cases of reactivation due to elective immunosuppression, effective treatment was available after temporary cessation of immunosuppressive drugs. In contrast, reactivation following successful treatment has not been described for *L. major* infection. Clinical experience thus supports use of *L. major* as a challenge agent, whilst highlighting a difficulty for developing CHIMs with other species. Counterbalancing risk, there is potential for CHIM participants to gain benefit from participation if traveling to a leishmaniasis endemic region through acquisition of immunity. Given development of lesions, treatment to terminate infection may itself carry risks. The risks of current *Leishmania* drug treatments are well established, although a proposal to carry out biopsy to terminate an infection has been approved as part of a proposed *Leishmania* CHIM [80]. Such a biopsy is likely to carry minimal risks and would be akin to performing a small diagnostic biopsy which is routine dermatological practice for many skin conditions.

Interspecies variation of *Leishmania* has also allowed for adaptation to different climates, with the implication being that some species have potential for spread to non-endemic settings [91]. The risk of introduction of *L. major* to non-endemic settings via a CHIM study is likely to be low, given the absence of natural vector. The risk may be increased in settings where other species of *Leishmania* and/or sand flies are present and anthroponotic transmission predominates, although not all species of *Leishmania* are transmissible by all species of sand fly. Similarly, the ethical and regulatory issues related to introducing non-native *Leishmania* species into an endemic country as part of a CHIM study would need to be balanced against the added value of conducting CHIMs in an endemic country setting and the risks of using parasite species where propensity for reactivation is less well understood.

### 3.5. Vector-specific factors

The study of the sand fly bite reaction itself in humans has taken place for close to a hundred years, with evolving understanding of its importance in establishing disease [85,92]. The importance of sand fly salivary gland protein alone has also been reinforced with the demonstration of protection against further challenge with *Leishmania* in mouse models [93,94].

Leishmaniasis is naturally transmitted by sand flies and sand fly salivary components as well as sand fly microbiota may contribute to parasite establishment and disease progression [93–95]. Importantly, vaccines effective against needle challenge may not always protect against natural challenge [96], indicating that vaccine development programs may be best served not solely by using humans as the model system but also by incorporating a natural route of challenge.

The sand fly has a complex relationship with both host and parasite which is difficult to replicate in settings where the sand fly is absent [2,97]. Given this multifaceted relationship between parasite, host and vector, the use of the sand fly vector is an important consideration in any proposed *Leishmania* CHIM. A key issue is however the extra layer of complexity that using vectors poses to deliver challenge agents. In addition to logistical issues related to sand fly colony maintenance, transport and/or co-location at the clinical site, vector transmission introduces issues of reproducibility of inoculum size and biting frequency that may impact significantly on the design and analysis of a clinical study. Although transmission of human infective *L*. (*Leishmania*) and *L*. (V*iannia*) species is restricted to phlebotomine sand flies, evidence suggests that non-phlebotomine vectors such as *Culicoides* [98] may play a role in the transmission of species belonging to the phylogenetically ancient *Leishmania* (*Mundinia*) subgenus. This may require adaptation of proposed CHIM models to ensure vaccine protection against non-phlebotomine initiated infection.

A study entitled FLYBITE, a precursor to a future *Leishmania* CHIM, formalized a working protocol for using sand fly biting on humans. This study compared biting rates of two vectors of *L. major, P. papatasi* and *P. duboscqi*, evaluating efficacy of the procedures and participant safety. The study had two arms, with six participants assigned to each vector, no placebo or control group was employed. Five sand flies were placed within a sand fly biting chamber that was worn for 30 minutes close to the antecubital fossa of the non-dominant arm. After completion of feeding, participants were monitored on site for 2 hours with subsequent follow-up until day 21. There was no significant difference between biting rates of both species, and each participant sustained at least one successful sand fly bite (mean 3.67 ± 1.03 bites per participant). There were no significant adverse effects reported, although participants reported expected and solicited events, including erythema and itch at the bite site. The experiences of participants were overwhelmingly positive, as evidenced by a post-study focus group. The study protocol and associated documents have been made available [99].

### 3.6. Parasite selection and manufacture

CHIM studies require the use of a well-characterized challenge agent to ensure a reproducible attack rate and mitigate against adverse effects. Although challenge agents are not regulated by the MHRA in the UK, informal regulatory advice emphasizes the need for challenge agents to have established provenance and for characterization of challenge agent donors for other transmissible disease (e.g. retroviruses). In addition, in vitro culture supplements should be from sources free of agents responsible for transmissible spongiform encephalopathies. Ashwin et al. have described in detail elsewhere a new *L. major* strain for use in controlled human infection research [100]. There are several existing parasite repositories that are used for experimental animal work. However, the provenance of the parasites they contain was insufficiently defined with minimal relevant clinical information from donors and/or relating to passage conditions. In addition, cultured *Leishmania* promastigotes may not be reliable in terms of their infectivity [101]. The new GMP produced clinical bank should suffice to serve the leishmaniasis vaccine development community for several years to come.

The use of *L. major* in vaccine research for leishmaniasis is of importance in two linked ways. Firstly, given that a breadth of clinical forms of leishmaniasis exist, it has been shown that *L. major* confers heterologous protection against species that cause visceral leishmaniasis [102]. Although the *Leishmania* genus shares a highly conserved genome, the relative phylogenetic distance between New World and Old World species for both cutaneous and visceral species is significant [103]. There is, however, some evidence for the potential for attenuated Old World species to provide heterologous protection against New World disease [104,105]. This adds further weight to the notion of a pan-species vaccine. Secondly given this cross protection, it is expected that a controlled human infection model using *L. major* will be informative for evaluating likely protection against other species of *Leishmania*. Additionally, *L. major* generally causes single lesions, which have the potential to self-heal, and with suitable treatment available to successfully terminate infection, in contrast with many other species. Importantly no evidence exists for reactivation of *L. major* with particular relevance to immunosuppression [102], and the disease course is thought to be benign in the overwhelming majority of individuals.

There are several novel attenuated parasite strains that may prove beneficial for use in a future CHIM, providing a possible route to using species that cause visceral disease. Attenuated strains, however, may show loss of virulence and therefore falsely reassure and conversely, depending on their mode of generation, may revert to wild type and therefore the potential for unexpectedly aggressive disease could emerge. A *L. major* knockout deficient in lipophosphoglycan, one of the primary targets at the host-parasite interface, caused no observable disease in mice but demonstrated persistent immunity to re-challenge with a virulent *L. major* strain [106]. An *L. donovani* centrin knockout strain, in tandem with a sand fly salivary gland protein conferred long-term protection in mice, suggesting possible use of needle challenge within a CHIM in the absence of a suitable vector [107]. An *L. infantum* HSP70 knockout has demonstrated heterologous protection following inoculation of mice, in both Old World and New World species [104,108]. Importantly a *L. major* centrin knockout produced using CRISPR technology has recently been shown to provide cross protection against fatal sand fly transmitted *L. donovani* infection in the hamster model [109].

Leishmaniaviruses (LRVs) are symbiotic RNA viruses found in several species of *Leishmania* [110]. The role of the LRVs in disease is still not well understood, although they may impact the disease course and be linked to increased severity [111,112], especially in New World species causing mucocutaneous disease such as *L. (V.) guyanensis* [113,114]. The use of New World *Leishmania* species infected with LRV is therefore problematic for CHIM studies, requiring LRV-free strains to be produced, which may not accurately reproduce all aspects of parasite behavior. Whilst LRV1 has been found in *L. major* [115] very little is known about its relationship to disease caused by this species. A small study in Iran did not determine a clear relationship between presence of LRV and treatment response in *L. major* infection [116]. Nevertheless, absence of LRV1 was confirmed for the new *L. major* strain used for GMP production [100].

Given the multiple challenges posed by using vectors within a CHIM study, there is a potential for using needle challenge, as has been used in multiple previous experimental *Leishmania* infections, if sand fly-initiated infection is unsuccessful. Although this has the disadvantage of lacking sand fly-related factors, it may allow for fewer logistical challenges, and in theory can be rolled out rapidly. The development of a parasite bank to GMP allows for this possibility if, for example, the use of sand fly initiated human experimental infection fails or is impractical. However, some additional considerations related to culture expansion and/or the isolation of infective metacyclic promastigotes prior to inoculation may have to be considered. CRISPR gene deletion technology also allows for attenuated strains to be used in such a scenario, particularly where editing genomes may impact on the transmission cycle using sand flies [117]. This possibility may also allow for future live attenuated parasite vaccination strategies and for the use of such attenuated parasites as challenge agents.

Although a *L. major* CHIM may have predictive value for a heterologous vaccine, a standalone visceral or mucocutaneous leishmaniasis model would be gold standard for these respective diseases. Proposing use of a VL-CHIM model could lead to discussions of the use of xenodiagnoses to determine infectiousness of participants [118]. As has been alluded to, this is not without significant consideration with respect to risk, in part due to persistence of parasites and subsequent transformation of cases to PKDL, difficulty of treatment options, and increased morbidity and mortality in comparison to cutaneous disease. Parasite persistence in visceral disease is also associated with recrudescence after initially quiescent disease, which can occur potentially many years downstream. This can be exacerbated after both primary and acquired immunodeficiency, and thus the follow-up period of 6 months that has been suggested for a CHIM for cutaneous leishmaniasis would not suffice for a visceral leishmaniasis model [80].

### 3.7. Study design considerations

An initial protocol for a CHIM study using the challenge strain described above has been developed (ClinicalTrials.gov: NCT04512742) [80]. This *Leishmania* CHIM is a clinical study in up to 18 healthy *Leishmania*-naïve participants. Initially, participants will be exposed to biting by *P. duboscqi* infected with *L. major*. An adaptive design is proposed to minimize unnecessary exposure of participants to *Leishmania* and maximize the likelihood of developing a reproducible CHIM. All participants will be continually reviewed for development of CL lesion and when reaching 3 mm diameter, this will be removed by excision biopsy. Should a subsequent lesion develop after excision the participants will be referred to clinical specialists for ongoing management. Following completion of the study procedures, a focus group will take place. As the aim of this study is to test efficacy of the challenge model, i.e. reproducibility of sand fly-initiated infection, an ideal situation would be if all patients in the initial cohort develop lesions within the 6-month post-challenge phase of the study. If take rate is lower, however, modifications to the protocol for example, a switch to an alternate vector such as *P. papatasi* would be considered.

It may be valuable to evaluate new vaccine candidates additionally in an endemic country-based CHIM, to more accurately represent the at-risk population, though the number of permutations of parasite species and sand fly vector needed to cover all geographies is daunting. CHIMs might need to be modified to account for variations in differing susceptibility and transmission efficacy of vectors and parasite in different ethnicities. An established CHIM used in endemic settings could show different transmission dynamics in non-naïve participants. Even where participants have not been exposed to *Leishmania*, there is a reasonably high probability of exposure to sand fly bites in endemic regions and therefore salivary gland proteins and other sand fly-derived factors. These may attenuate disease course and impact on the outcomes of any vaccine study both positively and negatively [93].

## 4. Expert opinion

The development of an effective CHIM for cutaneous leishmaniasis has the potential to significantly improve the timeline for development of vaccines already in the pipeline and stimulate discovery research on vaccines by providing a clear route to efficacy studies in humans. Hence, research on CHIMs for leishmaniasis can contribute to disease control efforts, and impact morbidity and mortality [119]. It remains to be determined whether CHIM studies will be able to replace conventional efficacy trials, as mooted for pandemic diseases [120], but even if this is not the case, they are likely to guide decisions as to which candidates enter costly large-scale vaccine studies. As with other diseases, a leishmaniasis CHIM will also provide an opportunity to gain new insights into the early stages of leishmaniasis disease progression. Application of deep phenotyping methodology, including transcriptomics, metabolomics and proteomics to both blood and tissue may identify new pathways associated with both natural and vaccine-induced resistance, and help to determine in a more directly comparative manner, the true values of current experimental models of infection.

Given the complexity of the pathogenesis of leishmaniasis, the broad spectrum of disease and the nature of CHIM studies, some outstanding questions remain. Experimental human infection using species that cause visceral leishmaniasis has been used previously [50], although even with current treatments and understanding of disease course, this is unlikely to be part of future challenge studies. Nevertheless, within appropriate ethical boundaries and driven by clinical need, many modifications of the simple CHIM protocol could be envisaged. For example, future CHIM studies may wish to address the impact of repeated exposure to uninfected sand flies prior to human infection.

A first clinical study to evaluate the reproducibility of a CHIM for sand fly transmitted cutaneous leishmaniasis has gained favorable ethical and institutional approval [80], with recruitment due to commence shortly [121]. If successful, it is very likely that in the coming years, CHIMs will become incorporated into the pipeline for candidate vaccines for leishmaniasis. It is acknowledged that in many arthropod-borne diseases such as leishmaniasis, vector-specific factors are important for establishing infection and therefore for determining vaccination strategy. Success of a CHIM for leishmaniasis may encourage researchers to facilitate research underpinning the use of other *Leishmania*-transmitting vectors, but also the consideration of CHIMs in other biting arthropod-driven diseases. An important question, however, for any CHIM as well as future similar models, is the extrapolation of vaccine efficacy derived from these models. This is a challenging question and will not be answered until retrospective analysis following widespread testing of candidate vaccines.

The COVID-19 global pandemic is likely to have far-reaching consequences for research funding for neglected tropical diseases as well as disease control. Modeling has demonstrated that delays in the visceral leishmaniasis elimination target in India are probable, and the number of new cases are likely to rise accordingly [122]. A successful CHIM is likely to dramatically affect the field, reducing time to market for vaccines, giving greater understanding of disease at the host-pathogen-vector interface, and improving cost-effectiveness of vaccine development.

## Article highlights

- Leishmaniasis control has been challenging, contributed to by a lack of an effective human vaccine and limited drug therapies.
- Controlled human infection models have demonstrated efficacy of vaccine candidates at an early stage of development. Where vaccine candidates have not demonstrated success using such models, such studies have contributed to new concepts that underpin understanding of disease pathogenesis.
- Deliberate human infection using *Leishmania* parasite has been carried out for several centuries in a practice known as leishmanization. Individuals in endemic areas were inoculated with live parasites that prevented further infection after subsequent re-exposure.
- Over the past century, deliberate exposure to *Leishmania* has been used to study methods of transmission as well as the immunology of disease at the host-vector-parasite interface.
- In any CHIM study it is imperative that a well-characterized infectious challenge agent is employed. A parasite bank for *Leishmania major*, manufactured to GMP, has been developed. It is expected that this parasite bank will be large enough to satisfy numerous CHIM studies in the future.
- Following demonstration of safety and reproducibility of uninfected sand fly bites and studies on the public perception of the study design, a CHIM protocol has received ethical approval in the UK.
- Currently, the majority of CHIM studies take place in non-endemic settings, later being transferred to endemic settings. This dynamic is challenging for a multitude of reasons. Future CHIMs may be developed directly in endemic settings, building long-term resources and infrastructure. The new CHIM for cutaneous leishmaniasis is expected to expedite the selection of promising new vaccines for progression to large-scale clinical trials.
